# Identification of Quantitative Trait Loci Controlling Root and Shoot Traits Associated with Drought Tolerance in a Lentil (*Lens culinaris* Medik.) Recombinant Inbred Line Population

**DOI:** 10.3389/fpls.2016.01174

**Published:** 2016-08-23

**Authors:** Omar Idrissi, Sripada M. Udupa, Ellen De Keyser, Rebecca J. McGee, Clarice J. Coyne, Gopesh C. Saha, Fred J. Muehlbauer, Patrick Van Damme, Jan De Riek

**Affiliations:** ^1^Department of Plant Production, Faculty of Bioscience Engineering, Ghent UniversityGhent, Belgium; ^2^Institut National de la Recherche Agronomique du Maroc (INRA), Centre Régional de SettatSettat, Morocco; ^3^International Center for Agricultural Research in the Dry Areas, Institut National de la Recherche Agronomique Morocco Cooperative Research ProjectRabat, Morocco; ^4^Plant Sciences Unit, Applied Genetics and Breeding, The Institute for Agricultural and Fisheries Research (ILVO)Melle, Belgium; ^5^United States Department of Agriculture, Agricultural Research Service Grain Legume Genetics and Physiology ResearchPullman, WA, USA; ^6^United States Department of Agriculture, Agricultural Research Service Western Regional Plant Introduction, Washington State UniversityPullman, WA, USA; ^7^Brotherton Seed CompanyWashington, DC, USA; ^8^Faculty of Tropical AgriSciences, Czech University of Life SciencesPrague, Czech Republic

**Keywords:** lentil, ecophysiology, drought tolerance, breeding, plant, QTL, marker-assisted selection

## Abstract

Drought is one of the major abiotic stresses limiting lentil productivity in rainfed production systems. Specific rooting patterns can be associated with drought avoidance mechanisms that can be used in lentil breeding programs. In all, 252 co-dominant and dominant markers were used for Quantitative Trait Loci (QTL) analysis on 132 lentil recombinant inbred lines based on greenhouse experiments for root and shoot traits during two seasons under progressive drought-stressed conditions. Eighteen QTLs controlling a total of 14 root and shoot traits were identified. A QTL-hotspot genomic region related to a number of root and shoot characteristics associated with drought tolerance such as dry root biomass, root surface area, lateral root number, dry shoot biomass and shoot length was identified. Interestingly, a QTL (QRSratio_IX-2.30_) related to root-shoot ratio, an important trait for drought avoidance, explaining the highest phenotypic variance of 27.6 and 28.9% for the two consecutive seasons, respectively, was detected. This QTL was closed to the co-dominant SNP marker TP6337 and also flanked by the two SNP TP518 and TP1280. An important QTL (QLRN_III-98.64_) related to lateral root number was found close to TP3371 and flanked by TP5093 and TP6072 SNP markers. Also, a QTL (QSRL_IV-61.63_) associated with specific root length was identified close to TP1873 and flanked by F7XEM6b SRAP marker and TP1035 SNP marker. These two QTLs were detected in both seasons. Our results could be used for marker-assisted selection in lentil breeding programs targeting root and shoot characteristics conferring drought avoidance as an efficient alternative to slow and labor-intensive conventional breeding methods.

## Introduction

Lentil (*Lens culinaris* Medik.) is an important grain legume crop that is often grown in sustainable farming systems and for nutrition in the world. Its ability to enhance soil fertility through atmospheric nitrogen fixation allows substantial reduction in fertilizer use and significant production improvement in cereal-based cropping systems thanks to the benefits of rotation.

Lentil grains are a rich source of proteins and some important micronutrients such as iron and zinc (Grusak and Coyne, 2009; Thavarajah et al., 2011). Consumed as staple food in developing countries and as vegetarian dishes elsewhere, lentil grains are considered a very healthy food. The United Nations, in its 68^th^ General Assembly, declared 2016 as the International Year of Pulses (annual leguminous crops harvested for dry grains) in order to highlight the nutritional benefits of pulses as part of sustainable food production aimed towards food and nutrition security (FAO, 2015).

In the arid and semi-arid areas and also in the context of climate change and global warming, drought is one of the major constraints that can limit lentil production and cause substantial yield losses (Malhotra et al., 2004; Stoddard et al., 2006; Sarker et al., 2009). Developing cultivars with enhanced drought tolerance by conventional breeding often has limited success due to the complexity of this trait and the difficulties with finding reliable and suitable phenotyping methods. For example, well-developed root systems have been shown to be linked to drought tolerance as an avoidance mechanism guaranteeing plant productivity under water-limited conditions (Kashiwagi et al., 2005; Sarker et al., 2005; Verslues et al., 2006; Gaur et al., 2008; Vadez et al., 2008; Aswaf and Blair, 2012; Comas et al., 2013; Idrissi et al., 2015a,b). However, it is difficult to screen large numbers of accessions for these root traits using conventional methods. Thus, applying a marker-assisted selection for these traits would offer an interesting alternative in breeding programs targeting drought tolerance. As such, identifying and mapping DNA markers linked to genes controlling rooting patterns associated with drought tolerance will assist in reliable and efficient identification and development of tolerant cultivars. Several studies have shown that root traits are polygenically controlled, whereas they also identified related quantitative trait loci (QTLs) for different species such as maize (Ruta, 2008), common bean (Cichy et al., 2009; Aswaf and Blair, 2012), barley (Sayed, 2011), soybean (Brensha et al., 2012) and chickpea (Kashiwagi et al., 2014).

Lentil has a genome size of about 4 Gbp (Arumuganathan and Earle, 1991); several kinds of DNA markers have been developed and mapped, including RAPDs, ISSRs, AFLPs, SRAPs, SSRs, and SNPs (Eujayl et al., 1998; Rubeena et al., 2003; Hamwieh et al., 2005; Saha et al., 2010; Sharpe et al., 2013). Idrissi et al. (2015a) confirmed evidence of high genetic variability, high heritability and polygenic control of root and shoot characteristics. However, to our knowledge, no QTLs related to root traits have been reported for lentil to date. Thus, the objective of this study was to identify and map QTLs related to root and shoot traits associated with drought tolerance in a lentil recombinant inbred line population (RIL) as a promising step towards a marker-assisted selection approach. It also aimed to investigate the stability of detected QTLs by performing the analysis on two consecutive seasons.

## Materials and methods

### Plant materials

A recombinant inbred line (RIL) population developed from a cross between two contrasting parents, ILL6002 and ILL5888 (Saha et al., 2010), obtained from Fred J. Muehlbauer, USDA-ARS, Washington State University, Pullman, USA, was used in this study. The RIL population consisted of the two parents and 132 F_6–8_ lines. The lines were advanced to the F_6–8_ generation from individual F_2_using single seed descent. The ILL6002 parent is a vigorous line reported as drought tolerant and with a well-developed root system (Sarker et al., 2005; Singh et al., 2013). On the other hand, ILL5888 is a drought sensitive line and has a less-developed root system and vegetative biomass. The two parents also differ in disease resistance (Stemphylium blight), flowering and maturity time, seed diameter, 100-seed weight, growth habit and plant height (Saha et al., 2010, 2013).

### RIL root and shoot traits phenotyping and drought tolerance evaluation

This F_6–8_ population was previously characterized for root and shoot traits related to drought tolerance (Idrissi et al., 2015a). Briefly, the population was evaluated under greenhouse conditions for root and shoot traits associated with drought tolerance under two contrasting watering regimes (well-watered and progressive drought-stressed) using the standard nutrition solution EEG MESTSTOF 19-8-16 (4) for two consecutive growing seasons (2013 and 2014). A completely randomized block design with three replications was used. Four uniformly germinated seeds were planted in plastic pots (H 35 × D 24 cm) filled with fine perlite (diameter ≤ 2 mm) in order to be able to extract intact roots without damage (Day, 1991; Rabah Nasser, 2009). The initial moisture in all the pots of both watering regimes was 75% of field capacity. It decreased to about 22% for the drought-stressed regime where plants were watered only once in the beginning of the experiment, while it was maintained at 75% for the well-watered treatment by watering plants twice a week as described in Idrissi et al. (2015a). At 38 days after sowing, plants were carefully extracted without damage to the roots, then shoots and roots were separated into plastic bags. Washed roots were preserved in a refrigerator (4°C, 90% relative humidity) to avoid drying before being scanned using an EPSON Scan scanner. The images were then analyzed using Image J software (Abramoff et al., 2004) combined with Smart Roots software (Lobet et al., 2011). From the scanned images, taproot length (TRL; cm plant^−1^), average taproot diameter (TRD; mm plant^−1^), root surface area (RSA; cm^2^ plant^−1^) and lateral root number (LRN) were measured. Dry root and shoot biomass (DRW, DSW; mg plant^−1^) were measured after oven-drying at 72°C for 48 h. Chlorophyll content was estimated according to the SPAD values measured at 32 days after sowing using a SPAD-502Plus chlorophyll meter (Konica Minolta, Japan), four measures were taken in fully expanded leaves per plant. The wilting score (WS) corresponding to the degree of wilting severity was used to estimate drought tolerance using the following 0–4 score scale (Singh et al., 2013): 0 = healthy plants with no visible symptoms of drought stress; 1 = green plants with slight wilting; 2 = leaves turning yellowish green with moderate wilting; 3 = leaves yellow–brown with severe wilting; and 4 = completely dried leaves and/or stems. Seedling vigor (SV) was recorded following the 1–5 IBPGR and ICARDA (1985) scale: 1 = very poor; 2 = poor; 3 = average; 4 = good; 5 = excellent. Root–shoot ratio (RS ratio) was calculated by dividing the dry root weight by the dry shoot weight. Growth rate (GR; cm) was estimated as the gain of length between 12 (SL12DAS; cm) and 22 days after sowing (SL22DAS; cm; GR = SL22DAS–SL12DAS). Specific root length (SRL) and specific root surface area (SRSA) were estimated by dividing root length and root surface area, respectively, by dry root weight. All the measures were recorded as the mean value based on the four plants per individual genotype in each pot. A summary of genetic variation and heritability of these traits is provided in Table 4 (Supplementary Material).

### RIL genotyping

The previously developed linkage map of Saha et al. (2010) was created based on the same RIL population used in this study (ILL6002 × ILL5888). The initial mapping data of Saha et al. (2010) which consisted of 23 SSR, 108 SRAP, and 30 RAPD markers, were kindly provided by the authors. The map was further enhanced using 220 polymorphic Single Nucleotide Polymorphism (SNP) markers developed using the Genotyping By Sequencing (GBS) technique and 180 polymorphic Amplified Fragment Length Polymorphism (AFLP) markers.

### Genotyping by sequencing for SNP identification

SNP data were obtained from 92 (out of 132) RILs using GBS. The GBS procedure of Poland et al. (2012) was used, including their 48 bar-coded adapters with a *Pst I* overhang; genomic DNA was digested with the enzymes *Pst I* and *Msp I*. The ligation reaction was completed using bar-coded Adapter 1 and the common Y-adapter in a master mix of buffer, ATP and T_4_-ligase. Ligated samples were pooled and PCR-amplified in a single tube, producing libraries of 48 samples each. The libraries were sequenced on two lanes of Illumina HiSeq2000 (University of California Berkeley V.C. Genomic Sequencing Lab). The sequencing data were processed to remove low quality data using in-house scripts and analyzed using Stacks software (Catchen et al., 2011, 2013). Two hundred twenty SNPs that proved to be polymorphic between both parents of the RIL population ILL6002 × ILL5888 (Wong et al., 2015) were analyzed.

### AFLP genotyping

The AFLP protocol of Vos et al. (1995) with minor modifications (De Riek et al., 2001) was performed as described in Idrissi et al. (2015c). Out of 12 primer combinations tested, seven (*EcoRI-ACA* + *MseI-CAG, EcoRI-ACA* + *MseI-CTG, EcoRI-ACA* + *MseI-CTT, EcoRI-ACG* + *MseI-CAA, EcoRI-AGC* + *MseI-CAA, EcoRI-AGC* + *MseI-CAG, EcoRI-AGC* + *MseI-CTG*) were selected and used for genotyping the RIL population.

### Linkage analysis and map construction

Five hundred sixty-one molecular markers on 132 RILs were used for linkage analysis and construction of a linkage map using JoinMap®4 (Van Ooijen, 2006; Table 1). First, segregation according to Mendelian expectation ratio of 1:1 was tested using the chi-square test at a significance level of 0.05, markers with distorted segregation were removed prior to further analysis. The grouping tree of the JoinMap® program was calculated using independent *LOD* (Logarithm of odds) as grouping parameter with threshold ranges of 6 for start and 30 for end, and 1 for step. Stable sets of markers at higher LOD values were selected. After initial creation of groups, the *Strongest Cross Link* (*SCL*) information from the output results was used for inspecting assignment of markers to groups, those with *SCL-values* larger than 5, indicating that they have strong linkage outside their respective groups, were assigned to the corresponding groups. This was repeated until all markers of each group had *SCL-values* smaller than 5. Linkage groups were calculated using the maximum likelihood mapping algorithm with default values as in the software. Map order in each linkage group was verified using the regression mapping algorithm with the following parameters: *LOD* threshold larger than 4, recombination frequency smaller than 0.25, Kosambi function as mapping function for genetic distance calculation and the second round map of the algorithm. The final linkage map was generated using MapChart^©^ 2.3 program (Voorrips, 2002).

**Table 1:** **Marker types used for linkage map development**.

**Marker types**	**Number of polymorphic markers**	**Final number of mapped markers**
SNP	220	106
SSR	23	13
SRAP	108	56
AFLP	180	60
RAPD	30	17

### QTL analysis

QTL analysis was performed for each season separately for drought-stressed treatments in order to check the stability of detected QTLs using MapQTL® 5 program (Van Ooijen, 2004). First, Kruskal-Wallis test was performed to determine a set of markers linked to each quantitative trait. Simple Interval Mapping was performed to identify linkage groups and positions with significant LOD scores. For each trait, LOD score threshold was determined based on a permutation test using 1000 iterations at a *P*-value of 0.05; LOD scores above these values were considered as significant. Co-factor selection was performed based on automatic co-factor selection implemented in the software for each linkage group and on manual selection of individual markers with significant LOD scores from Simple Interval Mapping output before applying Multiple-QTL Models (MQM) mapping (also called Composite Interval Mapping). Performing MQM mapping with markers close to significant LOD score positions as co-factors allows reduction of residual variance, thus enhancing the power of QTL detection. For each quantitative trait, co-factor selection and MQM mapping were repeated until no further enhancement was obtained (no more QTLs detected, increase in LOD scores and explained variances). From the MQM mapping output, closest marker, flanking markers, additive affect and percentage of explained variance for each detected QTL and for each quantitative trait were determined for seasons. Final results, with significant LOD scores and intervals, for each detected QTL per linkage group, were generated using MapChart^©^ 2.3 program (Voorrips, 2002).

All detected QTLs were named as follows: Q‘Trait name abreviation'_“linkage group number” − “position in cM”_. For example: QLRN_III−98.64_ is a QTL associated with LRN identified in linkage group III at position 98.64 cM.

## Results

### GBS for SNP identification

**Figure 1:**
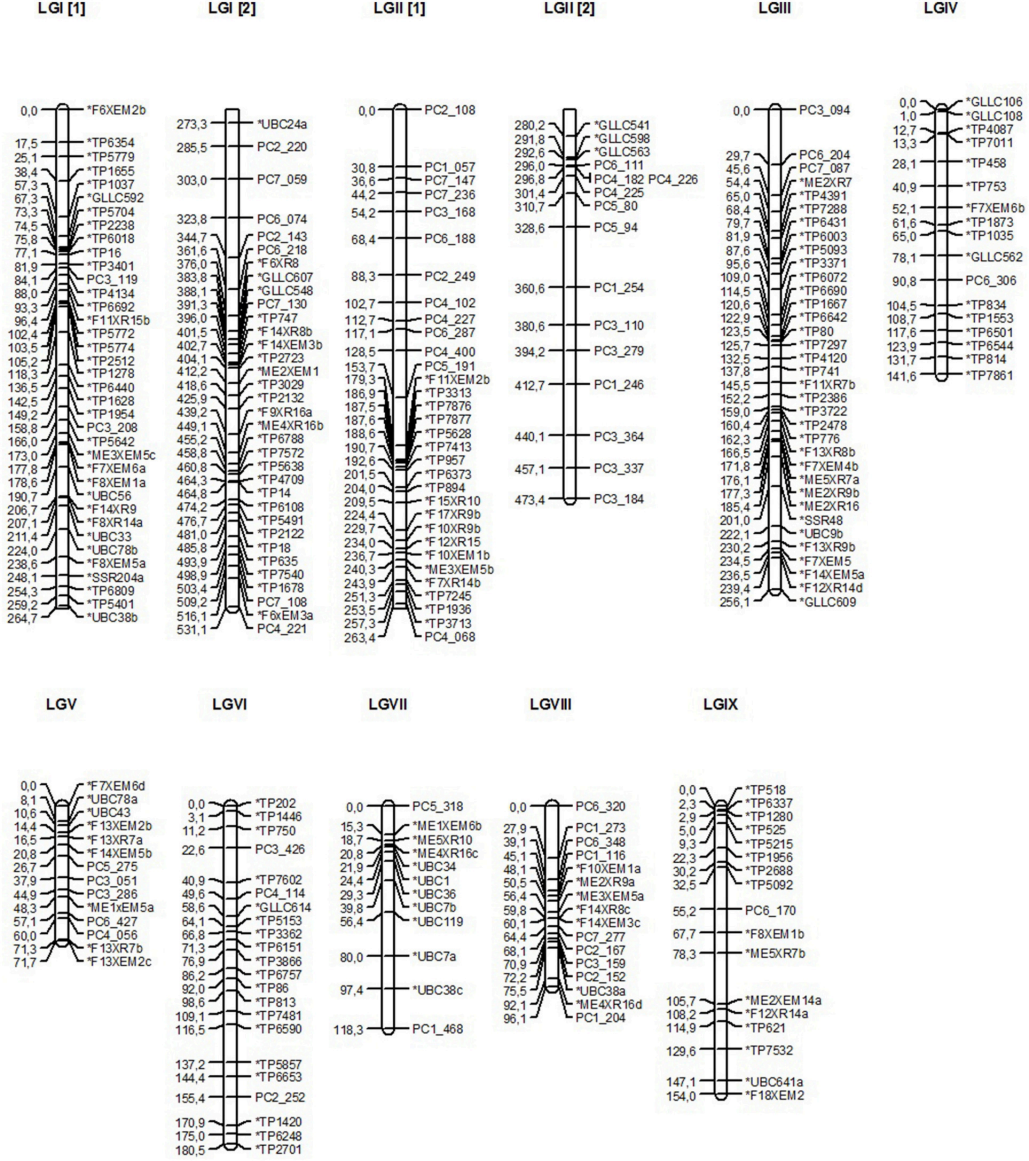
**Genetic linkage map of lentil developed at LOD score > 6 using maximum likelihood mapping algorithm of JoinMap® 4 program and drawn using MapChart^**©**^ program**. LGI-LGIX correspond to linkage groups, marker names are presented right to the linkage group and the genetic positions in CentiMorgans (cM) left. SNP markers are denoted by ^*^TP_, SSR by ^*^GLLC_, SRAP starting by ^*^M_ or F_, RAPD by ^*^UBC_ and AFLP by PC_.

Selection of the genotyping-by-sequencing two enzyme method of Poland et al. (2012) and the enzymes *Msp I* and *Pst I* was based on the results of Wong et al. (2015) lentil SNP discovery across the lentil species. Using GBS, 220 polymorphic SNPs were deemed high quality for mapping, after satisfying quality control filtering based on deleting low quality and redundant SNPs using haplotype information for read depth (3), lack of redundancy and segregation in the parents. Genome coverage was reasonable, but incomplete, across six linkage groups (LG I, LG II, LG III, LG IV, LG VI, and LG IX; Figure 1).

### Linkage analysis and map construction

Marker distortion tested by Chi-square test (*P* < 0.05) revealed that 35.4% of SNPs, 43% of SSRs, 18% of SRAPs, 52.7% of AFLPs and 20% of RAPDs did not segregate according to the expected 1:1 ratio and were removed from the analysis. Out of 17 stable groups selected from the grouping tree, a total of 252 out of the 561 polymorphic markers were finally mapped in nine linkage groups spanning a total length of 2022.8 cM (Tables 1, 2). Final linkage groups were established using the *SCL* information. Linkage group length ranged from 71.7 to 531.1 cM whereas average distance between two markers ranged from 5.12 (LG V) to 9.8 cM (LG II) (Table 2; Figure 1). Seven linkage groups had a length of more than 100 cM (LG I, LG II, LG III, LG IV, LG VI, LG VII, and LG IX). Both co-dominant (SNP and SSR) and dominant (SRAP, AFLP, and RAPD) markers were present in six linkage groups, while three linkage groups (LG V, LG VII, and LG VIII) were composed only out of dominant markers.

**Table 2:** **Linkage groups of the developed lentil linkage map and marker distribution**.

**Linkage groups**	**Number of mapped markers**	**Length (cM)**	**Average distance between markers (cM)**
LGI	71	531.1	7.4
LGII	48	473.4	9.8
LGIII	35	256.1	7.3
LGIV	17	141.6	8.3
LGV	14	71.7	5.12
LGVI	22	180.5	8.2
LGVII	12	118.3	9.8
LGVIII	16	96.1	6.0
LGIX	17	154.0	9.0
Total	252	2022.8	8.0

### QTL identification

**Figure 2:**
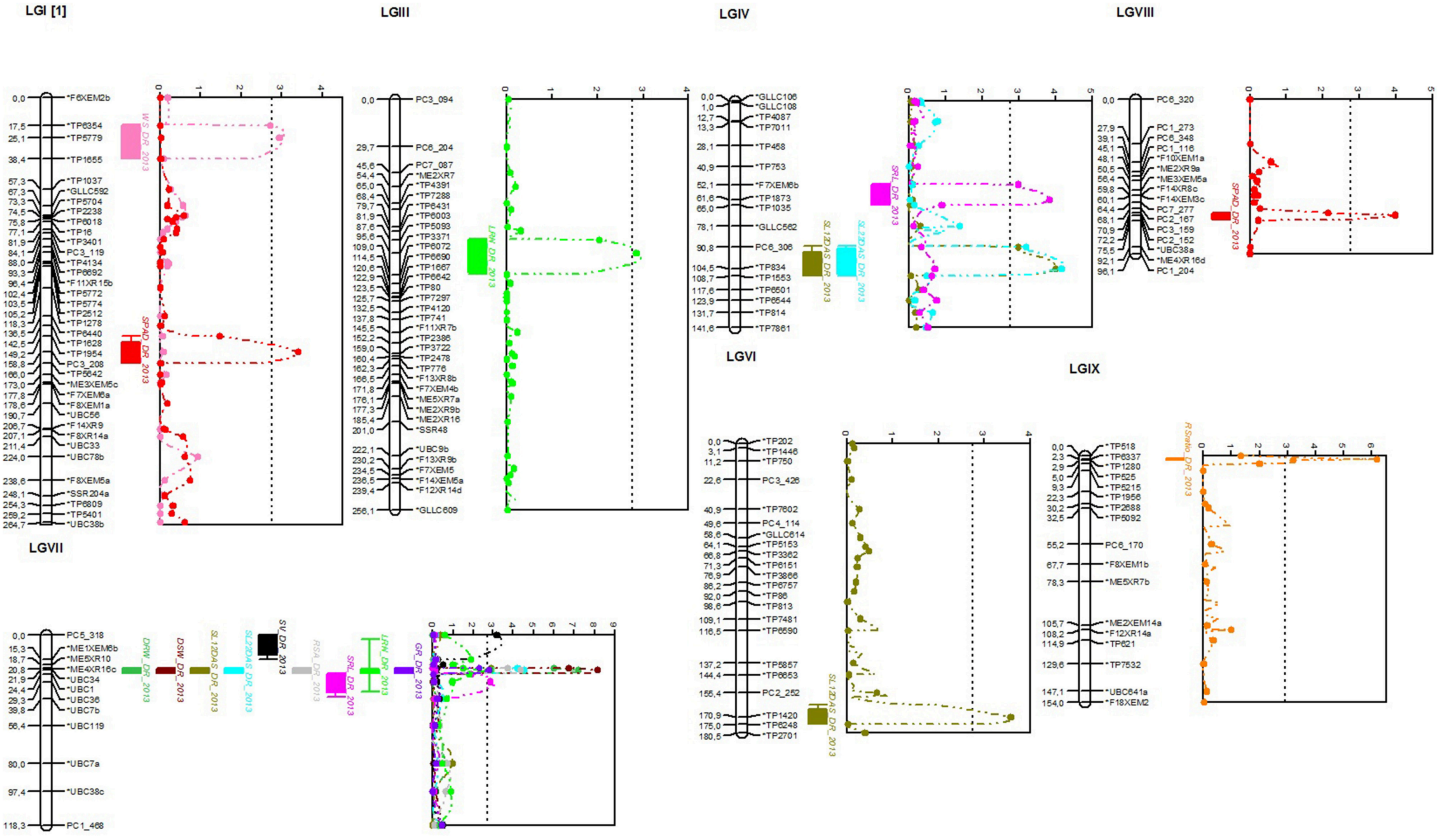
**Linkage groups with identified QTLs related to root and shoot traits under progressive drought stress for the 2013 season detected by MapQTL® 5 program and drawn using MapChart^**©**^ program**. LOD score curves are presented right to linkage groups and significant thresholds are presented by dotted lines (when more than one QTL for different quantitative traits are detected in same position, the dotted line correspond to the smallest threshold value). WS_DR_2013, wilting score; SPAD_DR_2013, chlorophyll content as estimated by SPAD value; LRN_DR_2013, lateral root number; SL12DAS_DR_2013, shoot length at 12 days after sowing; SL22DAS_DR_2013, shoot length at 22 days after sowing; SRL_DR_2013, specific root length; DRW_DR_2013, dry root weight; DSW_DR_2013, dry shoot weight; SV_DR_2013, seedling vigor; RSA_DR_2013, root surface area; GR_DR_2013, growth rate; RSratio_DR_2013, root-shoot ratio.

**Figure 3:**
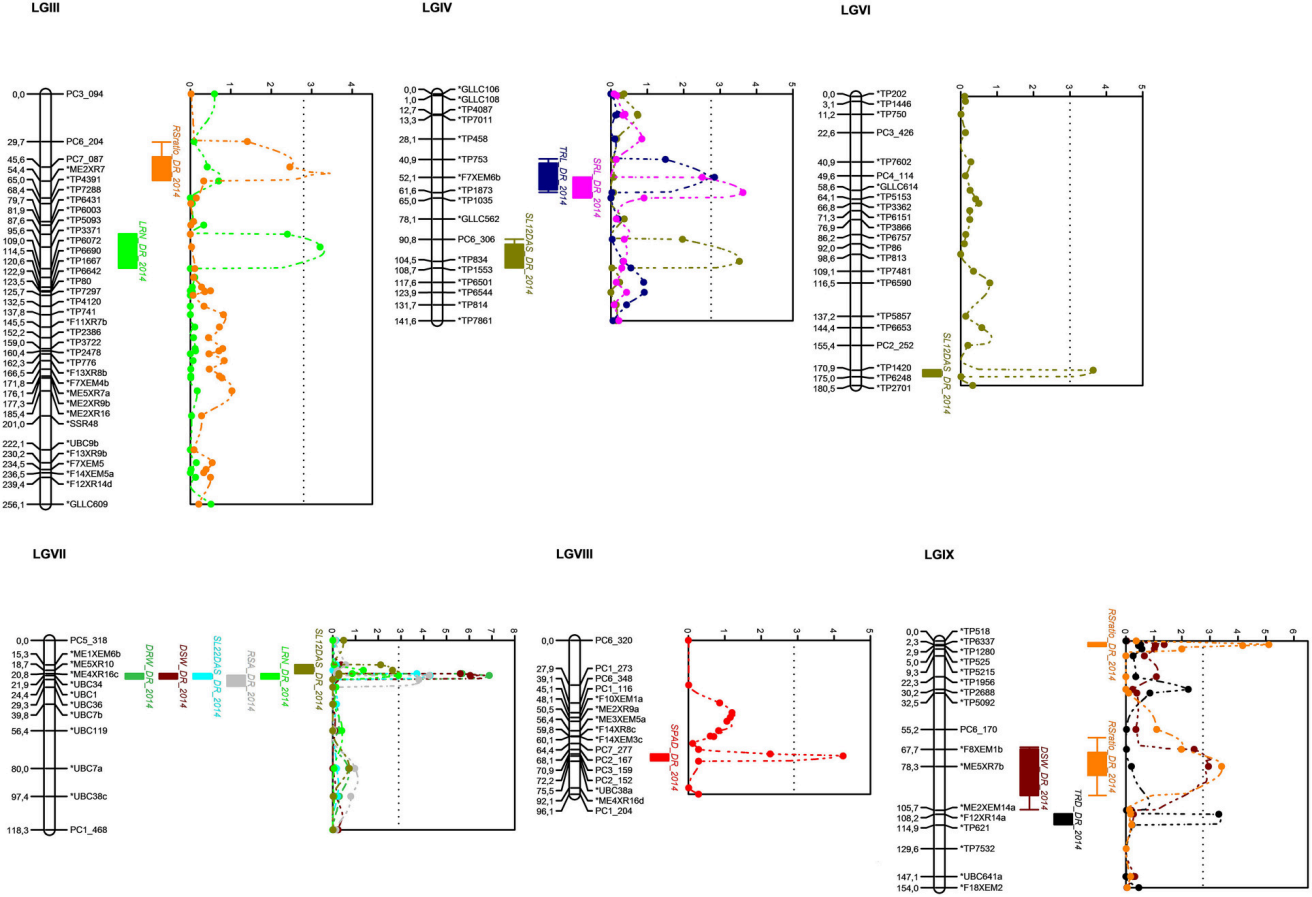
**Linkage groups with identified QTLs related to root and shoot traits under progressive drought stress for the 2014 season detected by MapQTL® 5 program and drawn using MapChart^**©**^ program**. LOD score curves are presented right to linkage groups and significant thresholds are presented by dotted lines (when more than one QTL for different quantitative traits are detected in same position, the dotted line correspond to the smallest threshold value). RSratio_DR_2014, root-shoot ratio; LRN_DR_2014, lateral root number; SRL_DR_2014, specific root length; TRL_DR_2014, taproot length; SL12DAS_DR_2014, shoot length at 12 days after sowing; DRW_DR_2014, dry root weight; DSW_DR_2014, dry shoot weight; SL22DAS_DR_2014, shoot length at 22 days after sowing; RSA_DR_2014, root surface area; SPAD_DR_2014, Chlorophyll content as estimated by SPAD value; RSratio_DR_2014, root-shoot ratio; TRD_DR_2014, average taproot diameter.

A total number of 18 QTLs associated with 14 root and shoot traits were detected under drought-stressed conditions during two seasons (Table 3; Figures 2, 3). LOD score, percentage of explained phenotypic variance and additive effect of detected QTLs ranged from 2.75 (TRL) to 8.14 (DSW), from 4.3 (QRSrati_IX-77.72_) to 28.9% (QRSratio_IX-2.30_) and from −5.17 (LRN) to 8.10 (DRW), respectively.

**Table 3:** **Characteristics of quantitative trait loci (QTL) identified under progressive drought stress in the RIL population (ILL6002 × ILL5888) for the 2013 and 2014 seasons**.

Trait^*^	QTL^**^	LOD Score ^***^	PVE% ^****^	Additive effect ^*****^	**Closest marker**	**Flanking markers**
**DROUGHT-STRESSED TREATMENT (2013)**						
DRW	**QDRW_VII−21.94_**	7.21	22.2	8.10	UBC34	ME5XR10 - UBC1
LRN	**QLRN_III−98.64_**	2.94	23.5	−5.17	TP3371	TP5093 - TP6072
	**QLRN_VII−21.94_**	2.91	5.3	2.78	UBC34	PC5_318 - UBC36
RSA	**QRSA_VII−21.94_**	4.36	14.1	1.68	UBC34	ME5XR10 - UBC1
DSW	**QDSW_VII−21.94_**	8.14	25.7	5.18	UBC34	ME5XR10 - UBC1
RS ratio	**QRSratio_IX−2.30_**	6.20	27.6	1.23	TP6337	TP518 - TP1280
SRL	**QSRL_IV−61.63_**	3.84	16.8	0.83	TP1873	F7XEM6b - TP1035
	QSRL_VII-31.25_	2.83	10.2	−0.03	UBC36	ME4XR16c - UBC7b
SL12DAS	**QSL12_IV−102.83_**	4.02	16.5	−0.54	TP834	PC6_306 - TP1553
	**QSL12_VI−170.87_**	3.58	15.9	0.50	TP1420	PC2_252 - TP6248
	**QSL12_VII−20.75_**	2.90	8.1	0.38	ME4XR16c	ME5XR10 - UBC34
SL22DAS	**QSL22_VII−21.75_**	4.55	12.2	0.78	UBC34	ME5XR10 - UBC1
	QSL22_IV-102.83_	4.22	19.2	−0.94	TP834	PC6_306 - TP1553
GR	QGR_VII-21.94_	2.82	9.4	0.41	UBC34	ME5XR10 - UBC1
SV	QSV_VII-4_	3.46	14.9	0.29	PC5_318	PC5_318 - ME1XEM6b
SPAD	**QSPAD_VIII−72.15_**	3.98	10.7	−2.20	PC2_152	PC3_159 - UBC38a
	QSPAD_I−158.76_	3.41	9.2	2.14	PC3_208	TP1954 - TP5642
WS	QWS_*I*-22.53_	3.08	18.8	0.46	TP5779	TP6354 - TP1655
**DROUGHT-STRESSED TREATMENT (2014)**						
DRW	**QDRW_VII−21.93_**	6.88	21.3	7.44	UBC34	ME5XR10 - UBC1
LRN	**QLRN_III−98.64_**	3.31	24	−5.15	TP3371	TP5093 - TP6072
	**QLRN_VII−21.94_**	2.89	10	6.98	UBC34	ME5XR10 - UBC1
RSA	**QRSA_VII−21.94_**	4.24	13.8	1.54	UBC34	ME5XR10 - UBC36
DSW	**QDSW_VII−22.94_**	6.96	20.7	4.40	UBC34	ME5XR10 - UBC1
	QDSW_IX-73.72_	2.90	9	−2.89	ME5XR7b	F8XEM1b - ME2XEM14a
RS ratio	**QRSratio_IX−2.30_**	5.11	28.9	1.84	TP6337	TP518 - TP1280
	QRSrati_IX-77.72_	3.45	4.3	0.14	ME5XR7b	F8XEM1b - ME2XEM14a
	QRSratio_III-49.62_	2.95	14.7	−0.14	PC7_087	PC6_204 - ME2XR7
TRL	QTRL_IV-52.11_	2.75	9.4	1.44	F7XEM6b	TP753 - TP1873
SRL	**QSRL_IV−61.63_**	3.63	16.2	0.32	TP1873	F7XEM6b - TP1035
TRD	QTRD_IX-111.15_	3.39	12.9	−0.04	F12XR14a	ME2XEM14a - TP621
SL12DAS	**QSL12_VI−170.87_**	3.64	15.8	−0.48	TP6248	TP1420 - TP2701
	**QSL12_IV−103.83_**	3.55	13.5	−0.45	TP834	PC6_306 - TP1553
	**QSL12_VII−19.71_**	2.94	8.1	0.35	ME5XR10	ME1XEM6b - ME4XR16c
SL22DAS	**QSL22_VII−21.94_**	4.28	12.1	0.65	UBC34	ME5XR10 - UBC1
SPAD	**QSPAD_VIII−72.15_**	4.25	13.1	−2.20	PC2_152	PC3_159 - UBC38a

* *DRW, dry root weight (mg plant^−1^); LRN, lateral root number; TRL, taproot length (cm plant^−1^); SRL, specific root length (cm mg^−1^ plant^−1^); TRD, average taproot diameter (mm plant^−1^); RSA, root surface area (cm^2^ plant^−1^); DSW, dry shoot weight (mg plant^−1^); SL12DAS, shoot length at 12 days after sowing (cm plant^−1^); SL22DAS: shoot length at 22 days after sowing (cm plant^−1^); GR, growth rate (cm plant^−1^); SV, seedling vigor; SPAD, chlorophyll content; RS ratio, root-shoot ratio; WS, wilting score*.

** *Bolded and underlined QTLs were identified for both seasons*.

*** *The presence of QTL was declared when the LOD score is above the threshold value obtained by a permutation test for each quantitative trait*.

**** *PVE: percentage of variance explained*.

***** *Positive values of additive effect mean that positive allele comes from the ILL6002 parent, while negative values mean that positive allele comes from the ILL5888 parent*.

Seven of the detected QTLs were co-located on LG VII at position 21–22 cM, with UBC34 as the closest marker and ME5XR10—UBC1 as the two flanking markers: QDRW_VII-21.94_, QLRN_VII-21.94_, QRSA_VII-21.94_, QDSW_VII-21.94_, QSL12_VII-20.75_, QSL22_VII-21.75_, and QGR_VII-21.94_.

Among the 18 detected QTLs, 12 were evidenced for the drought-stressed treatment for both seasons: QDRW_VII-21.93_, QRSA_VII-21.94_, QDSW_VII-22.94_, QRSratio_IX-2.30_, QSL12_IV-103.83_, QSL12_VI-170.87_, QSL12_VII-19.71_, QSL22_VII-21.94_, QLRN_III-98.64_, QLRN_VII-21.94_, QSRL_IV-61.63_, and QSPAD_VIII-72.15._ Interestingly, among these stable QTLs,QRSratio_IX-2.30_, located at 2.30 cM on LG IX, is associated with a high root-shoot ratio and had LOD scores of 6.20 and 5.11 for 2013 and 2014 seasons, respectively. The explained phenotypic variance of this QTL was the highest with 27.6 and 28.9% and an additive effect of 1.23 and 1.84 for 2103 and 2014 seasons, respectively. The closest marker to this QTL is SNP marker TP6337 located at 2.3 cM whereby the two flanking markers are TP518 and TP1280, located respectively at 0 and 2.9 cM.

Two QTLs were identified for dry root biomass, QDRW_VII-21.93_, accounted for 22.2% (with a LOD score of 7.21) and 21.3% (with a LOD score of 6.88) of the phenotypic variance with additive effects of 8.10 and 7.47 for 2013 and 2014 seasons, respectively.

Among the three QTLs detected for LRN, QLRN_III-98.64_, was located at 98.64 cM position on LG III close to TP3371 SNP marker and flanked by the two SNP markers TP5093–TP6072. The LOD scores, percentage of explained phenotypic variances and additive effects were 2.94, 23.5% and −5.17, and 3.31, 24%, and −5.15 for 2013 and 2014 seasons, respectively. An important QTL was also identified for SRL, namely QSRL_IV-61.63_, that was detected for both seasons with LOD scores, percentage of explained phenotypic variances and additive effects of 3.84, 16.8% and 0.83 and 3.63, 16.2% and 0.32, respectively, for 2013 and 2014.

Three QTLs were identified to be linked to chlorophyll content in which one was common for both seasons. The latter is the QTL QSPAD_VIII-72.15_, which was detected with LOD scores, percentage of explained phenotypic variances and additive effects of respectively 3.98, 10.7% and −2.20 for 2013 season and 4.25, 13.1% and −2.20 for 2014 season.

Also, a QTL related to early vegetative vigor estimated by SV was detected for the 2013 experiment. This QTL, QSV_VII4_, was located on LG VII at position 4 cM, had a LOD score of 3.46, an additive effect of 0.29 and explained 14.9% of total phenotypic variance.

A QTL QWS_I-22.53_, related to drought tolerance as estimated by WS, is located at 22.53 cM position on LG I with a LOD score of 3.08 and 18.8% as percentage of explained phenotypic variance.

## Discussion

The genetic linkage map of lentil initially developed by Saha et al. (2010) using a ILL6002 × ILL5888 RIL population containing 139 markers and 14 linkage groups was enhanced by adding SNP co-dominant markers and AFLP dominant markers, thereby increasing marker density and total spanned length. The number of linkage groups was reduced to nine with a total number of 252 mapped markers covering 2022.8 cM compared to 1565.2 cM in the previous genetic map. Average distance between markers was reduced from 11.3 to 8 cM. Sharpe et al. (2013) reported a lentil map with seven linkage groups using SNP and SSR markers. 62.77% of markers from the Saha et al. (2010) linkage map were also mapped in the genetic map developed in our study. Several sets of markers from the previous genetic map were confirmed to be linked to each other in our map. For instance, all markers from LG 1 from the map of Saha et al. (2010) were also mapped in LG I of our map. Thirteen markers out of a total of 19 mapped in LG 2 were mapped in LG III and four in LG IV of our map. Nine markers from LG 3 were mapped in LG V of our map and all those from LG 4 except for two that ended up in LG VII of our enhanced map. All markers from LG 11 of the previous map (except two) were mapped in LG II. All markers of LG 13 and LG 14 were mapped in LG IX and LG VIII of our map, respectively. Our linkage groups could not be assigned per Sharpe et al. (2013), the best lentil linkage map with seven linkage groups likely corresponding to the seven chromosomes of the genome developed to date, due to lack of common markers. We used a combination of dominant and co-dominant markers to develop a linkage map with reduced gaps. Since SNP data were not available for the whole population, we added also dominant AFLP markers for map construction. In other studies, dominant markers were also used together with co-dominant ones for the development of linkage maps and QTL analysis to overcome different limits such as genetic marker availability and large gaps in linkage groups (Gaudet et al., 2007; De Keyser et al., 2010; Kaur et al., 2014; Muys et al., 2014; Ting et al., 2014). Although, maximum likelihood mapping algorithm often results in increased map length, it is considered to be more robust with missing data, genotyping errors and the use of markers with low information content (Lincoln and Lander, 1992; Van Ooijen, 2006; Cartwright et al., 2007; De Keyser et al., 2010). This algorithm uses multipoint analysis to approximate missing genotypes using nearby markers (De Keyser et al., 2010). Genetic linkage maps based on this approach giving the most likely marker order (De Keyser et al., 2010) were reported to be suitable for QTL mapping (Kim, 2007; De Keyser et al., 2010). Thus, we adopted this approach as the main objective of our study was to identify QTLs related to root and shoot traits. Furthermore, although we used dominant markers such as AFLPs known to result in longer map, our linkage groups did not have extreme lengths and the total map length of 2022.8 cM is among common reported values in similar studies on lentil. Duran and Perez De La Vega (2004) reported a genetic linkage map of 2172 cM length using SSR, AFLP, ISSR and RAPD markers. Gupta et al. (2012) used SSR, ISSR and RAPD markers to construct a map of 3843.4 cM length. Also, Kaur et al. (2014) used SSR and SNP markers to develop a map of 1178 cM length. Using predominantly SNP markers and few SSRs, Sharpe et al. (2013) constructed a shorter map of 834.7 cM length. More recently, Ates et al. (2016) developed a map spanning a total length of 4060.6 cM and composed of seven linkage groups using SSR and SNP markers to identify QTLs controlling genes for Selenium uptake in lentil.

High genetic variability, quantitative, continuous and normally distributed variation as well as high heritability estimate values of all studied traits were reported in Idrissi et al. (2015a).

In all, 18 QTLs were identified for root and shoot traits for both seasons under progressive drought-stressed treatments in the lentil RIL population ILL6002 × ILL5888. Among these QTLs, 12 were evidenced for both seasons. Aswaf and Blair (2012) reported a total of 15 putative QTLs for seven rooting pattern traits and four shoot traits under drought-stressed treatments in common bean (*Phaseolus vulgaris* L.). Varshney et al. (2014) reported drought tolerance-related root trait QTLs in chickpea (*Cicer arietinum* L.). In soybean (*Glycine max* L.), Manavalan et al. (2015) identified a QTL region controlling a number of root and shoot architectural traits. In lentil, to our knowledge, this is the first report on QTLs related to root and shoot traits associated with drought tolerance. Interestingly, QTL QRSratio_IX−2.30_ related to root-shoot ratio, an important trait for drought avoidance (Verslues et al., 2006), was confirmed to be present on LG IX at 2.30 cM position during the two seasons. Among detected QTLs, this QTL explained the highest percentage of phenotypic variance and was close to the co-dominant SNP marker TP6337 (C/T) and furthermore was flanked by the two SNP markers TP518 (A/G) and TP1280 (G/T). These markers are potentially important for their practical use for marker-assisted selection in breeding programs targeting drought tolerance. It should be pointed out that the same SNP markers were confirmed as being linked to root-shoot ratio when using only SNP markers on 92 RILs for linkage map construction and QTL mapping (data not shown).

A QTL-“hotspot” genomic region was identified on LG VII close to UBC34 RAPD marker and ME4XR16c SRAP marker, and was identified to be linked to the genetic control of a number of root and shoot traits for both seasons: DRW, LRN, RSA, DSW, and SL at 12 and 22 days after sowing. These traits were shown to be significantly correlated (Idrissi et al., 2015a). Similarly, a QTL-“hotspot” related to 12 root traits was reported by Varshney et al. (2014) in chickpea (*Cicer arietinum* L.). Although practical efficient use of the identified genomic region in the ILL6002 × ILL5888 lentil population for marker-assisted selection could be limited by the dominant character of the closest RAPD marker, SRAP markers identified close to this genomic region could be used for assisting in the selection for linked traits. SRAP markers targeting the coding regions of open-reading frames of the genome, considered as better than RAPDs and technically less challenging than AFLPs, are of potential interest for QTL mapping (Chen et al., 2007; Yuan et al., 2008; Zhang et al., 2009; Saha et al., 2010, 2013; Robarts and Wolfe, 2014). Furthermore, up to 20% of SRAP markers were found to be co-dominant (Li and Quiros, 2001). Dry root weight, reported to be associated with drought tolerance by Idrissi et al. (2015a) in lentil, and other root and shoot traits such as root surface area and dry shoot weight also associated with drought tolerance are linked to this “hotspot” genomic region.

QTL QLRN_III−98.64_, related to LRN located at 98.64 cM position on LG III, was identified during both seasons explained 23.5 and 24% variations for 2013 and 2014, respectively. This QTL was close to SNP marker TP3371 (C/T) whereas its significant interval is between TP5093 (C/T) and TP6072 (A/G) SNP markers. Thus, the efficient use of these markers in breeding programs is possible for screening for higher LRN. High lateral root number was previously reported to be associated with drought tolerance and yield in lentil under drought stress (Sarker et al., 2005). Similarly, QTL QSRL_IV−61.63_, located at 61.63 cM on LG IV and related to SRL, was detected in both seasons with fairly high LOD scores of 3.84 and 3.63 for 2013 and 2014 respectively. This QTL, explaining 16.8% of phenotypic variance, was close to TP1873 (A/C) SNP marker (61.6 cM) and flanked by the two F7XEM6b SRAP (52.1 cM) and TP1035 (A/T) SNP markers (65 cM). Specific root length is considered an important root trait that can contribute to plant productivity under drought (Comas et al., 2013). Therefore, the use of these linked markers to screen lines with longer root length should be of potential interest. Three QTLs were identified for chlorophyll content as estimated by the SPAD value. Among them, QSPAD_I−158.76_ is located at 158.76 cM on LG I close to AFLP marker PC3_208 and flanked by the two co-dominant SNP markers TP1954 (A/T) and TP5642 (A/T) that could be efficiently used in marker-assisted selection. Idrissi et al. (2015a) reported correlations of SPAD value of 0.46 and 0.48 with dry root biomass and drought tolerance, respectively, in the same mapping population used here.

A QTL QWS_I−22.53_ related to drought tolerance as estimated by the WS, located at 22.53 cM on LG I and explaining 18.8% of total phenotypic variance, is close to TP5779 (A/T) and flanked by TP6354 (C/T) and TP1655 (C/T) SNP markers, was identified during the 2013 season. After validation, these markers could be used for screening for drought tolerance. Wilting severity due to drought stress was reported to be correlated with relative water content in lentil (Idrissi et al., 2015b) indicating the importance of this parameter for the identification of drought tolerant cultivars. QTLs for drought tolerance as estimated by relative water content were reported for pea (*Pisum sativum*) by Iglesias-García et al. (2015).

A drought tolerance breeding strategy could be first based on laboratory screening of large collections of genetic material for the presence of the identified markers. Then, lines carrying alleles linked to QTLs of targeted traits could be evaluated under field conditions to finally identify drought-tolerant individuals. More focus should be on QTLs related to root-shoot ratio, LRN, SRL, and WS shown to be flanked by SNPs markers. However, the QTL related to WS needs further evaluation under different watering conditions and drought intensity to determine environments of expression of this QTL. This will allow to determine whether it is an adaptive or constitutive QTL.

It should be pointed out that results of QTL analysis using the second round map of JoinMap®4 program (Van Ooijen, 2006) obtained from regression mapping algorithm were closely similar to those obtained using maximum likelihood algorithm, although total lengths of the two maps were different (data not shown).

## Conclusions

In this study, a total of 18 QTLs related to root and shoot traits associated with drought tolerance such as dry root biomass, LRN, root-shoot ratio, and specific root length were identified under progressive drought-stressed treatment. Interestingly, 12 of these QTLs were detected for both seasons, confirming their potential importance in conveying drought tolerance. DNA markers linked to these QTLs could be used for marker-assisted selection, thus making subsequent breeding efforts more reliable and efficient as the respective phenotyping-based methods are slow and labor-intensive, and affected by environment. Although, root characteristics are difficult to study as many environmental effects (especially soil characteristics) interact with genetic factors, our results provide significant information about QTLs related to root and shoot traits that could be used in marker-assisted breeding after validation.

## Author contributions

OI designed the study, analyzed data, interpreted results and wrote the paper. OI, JDR, PVD, EDK, SU contributed to design the study, analyze data, interpret results and wrote the paper. OI, CC, RM, GS contributed to data acquisition. SU, FM contributed to critically revising the paper.

### Conflict of interest statement

The authors declare that the research was conducted in the absence of any commercial or financial relationships that could be construed as a potential conflict of interest.
